# The Impact of Behavior Change Counseling Delivered via a Digital Health Tool Versus Routine Care Among Adolescents With Obesity: Pilot Randomized Feasibility Study

**DOI:** 10.2196/55731

**Published:** 2024-05-17

**Authors:** Maura Kepper, Callie Walsh-Bailey, Zoe M Miller, Min Zhao, Kianna Zucker, Angeline Gacad, Cynthia Herrick, Neil H White, Ross C Brownson, Randi E Foraker

**Affiliations:** 1 Prevention Research Center Brown School Washington University in St. Louis St. Louis, MO United States; 2 Institute for Informatics Washington University School of Medicine St. Louis, MO United States; 3 Division of Endocrinology Washington University School of Medicine St. Louis, MO United States; 4 Division of Pediatric Endocrinology & Diabetes Washington University School of Medicine St. Louis, MO United States

**Keywords:** digital health, obesity, clinical care, adolescents, physical activity, diet, clinical trial

## Abstract

**Background:**

Youth overweight and obesity is a public health crisis and increases the risk of poor cardiovascular health (CVH) and chronic disease. Health care providers play a key role in weight management, yet few tools exist to support providers in delivering tailored evidence-based behavior change interventions to patients.

**Objective:**

The goal of this pilot randomized feasibility study was to determine the feasibility of implementing the Patient-Centered Real-Time Intervention (PREVENT) tool in clinical settings, generate implementation data to inform scale-up, and gather preliminary effectiveness data.

**Methods:**

A pilot randomized clinical trial was conducted to examine the feasibility, implementation, and preliminary impact of PREVENT on patient knowledge, motivation, behaviors, and CVH outcomes. The study took place in a multidisciplinary obesity management clinic at a children’s hospital within an academic medical center. A total of 36 patients aged 12 to 18 years were randomized to use PREVENT during their routine visit (n=18, 50%) or usual care control (n=18, 50%). PREVENT is a digital health tool designed for use by providers to engage patients in behavior change education and goal setting and provides resources to support change. Patient electronic health record and self-report behavior data were collected at baseline and 3 months after the intervention. Implementation data were collected via PREVENT, direct observation, surveys, and interviews. We conducted quantitative, qualitative, and mixed methods analyses to evaluate pretest-posttest patient changes and implementation data.

**Results:**

PREVENT was feasible, acceptable, easy to understand, and helpful to patients. Although not statistically significant, only PREVENT patients increased their motivation to change their behaviors as well as their knowledge of ways to improve heart health and of resources. Compared to the control group, PREVENT patients significantly improved their overall CVH and blood pressure (*P*<.05).

**Conclusions:**

Digital tools can support the delivery of behavior change counseling in clinical settings to increase knowledge and motivate patients to change their behaviors. An appropriately powered trial is necessary to determine the impact of PREVENT on CVH behaviors and outcomes.

**Trial Registration:**

ClinicalTrials.gov NCT06121193; https://www.clinicaltrials.gov/study/NCT06121193

## Introduction

### Background

One-third of children and adolescents in the United States are classified as overweight or obese, with higher prevalence among racial and ethnic minority populations and those with low-income status [1]. The rise in pediatric obesity prevalence and severity brings with it the clustering of cardiometabolic risk factors such as hypertension, diabetes, dyslipidemia, chronic inflammation, and insulin resistance [2]. Overweight and obesity in adolescence is associated with greater risk of multimorbidity and mortality in adulthood [3]. Evidence-based interventions that improve physical activity, food intake, and BMI can prevent up to 40% of deaths [4]. The American Heart Association (AHA) has identified normal BMI, physical activity, and healthy food intake as critical for cardiovascular health (CVH) within its *Life’s Simple 7* metrics and the recently released *Life’s Essential* *8* [5,6]. Nevertheless, only 4% of adolescents meet the AHA *Life’s Simple 7* CVH metrics; this percentage is even lower among low-income adolescents who experience disproportionate barriers to optimal health, such as unmet social needs (eg, food insecurity and lack of transportation) [5,7-10].

Clinic-based interventions are a first-line approach to obesity prevention and management. Care teams have the potential to deliver health behavior counseling that motivates patients to achieve healthy behaviors [11,12]. Nevertheless, patients with obesity are advised to lose weight during only one-third of routine care encounters, and these discussions have demonstrated mixed effectiveness in generating behavior change [13]. The routine integration of evidence-based behavior change interventions in clinical care is currently lacking; a large gap remains between what is possible and what has been achieved [14]. While the US Preventive Services Task Force has recommended that ≥26 contact hours are necessary over 2 to 12 months to effectively intervene, subsequent studies have not demonstrated consistent hours-based dose-response and have shown that this amount of intervention is unrealistic for primary care or tertiary care providers [15]. Clinical care teams need further information on what can realistically be done within their contact hours to set these patients up for success [16].

Beyond time limitations, clinical care teams lack experience and confidence in navigating sensitive discussions, knowledge of evidence-based recommendations, or available resources (eg, time, technology, and supportive staff) to motivate and provide further contact hours and support for patients [17]. As outlined in the chronic care model for obesity management, engaging patients to be actively involved in their care is critical to achieving behavior change, supports patient autonomy and self-determination, promotes confidence and trust in the clinician-patient relationship, and improves satisfaction with care [18-20]. Nevertheless, health care teams do not have adequate web-based tools with interactivity, data visualization, and theory-driven evidence-based approaches to engage patients in setting behavior change goals [21,22]. The use of behavioral theory in such tools is critical to effectively promote behavior change [23]. The self-determination theory is a widely applied theory that may be integrated to help care teams increase patients’ intrinsic motivation to perform healthy behaviors by building autonomy, relatedness, and competence [24]. Ultimately, digital tools that use theory to facilitate efficient, meaningful, and patient-centered discussions and motivate patients could improve the effectiveness of health behavior counseling [13].

These discussions may be even more effective and reduce health disparities if they address the social and environmental context surrounding youth [25]. The ability to meet recommendations for behavior change (physical activity and healthy food intake) is influenced by the social and built environment [26-29], a lack of knowledge of existing resources, or limited infrastructure (eg*,* transportation) to access resources, particularly for racial and ethnic minority populations and those with low-income status [30-35]. Linking youth and their families to community resources aligns with the American Academy of Pediatrics recommendations for community pediatricians [36] and the chronic care model [20]. Several clinic-based interventions linking patients to community resources show promising weight loss results in adults and children [37-39]. Digital tools may support this type of referral with an interactional platform of community and digital resources accessible to the patient and shared among care teams to improve their awareness of resources and efficiency in providing support.

### Objectives

Digital tools may support this approach to improving health behavior counseling [40,41]. Digital tools can facilitate the collection of health behavior data (often unavailable at the point of care) and integrate it with electronic health record (EHR) data to generate an informed individually targeted intervention based on social and behavioral factors [42-44]. The use of data visualization has been shown to engage the patient, and interactional features can help facilitate shared decision-making [21]. Furthermore, digital tools provide platforms for patient communication that can increase the efficiency of regular check-ins with patients on their behavior change [21,45-47]. The Patient-Centered Real-Time Intervention (PREVENT) tool, described in detail elsewhere [48], was designed using the self-determination theory and with input from health care teams. PREVENT visually displays EHR and patient-reported CVH data, generates evidence-based tailored physical activity and nutrition goals, and includes a resource map and library to facilitate patient engagement in behavioral counseling. The goal of this pilot feasibility study was to determine the feasibility of implementing PREVENT in clinical settings, generate implementation data to inform scale up, and gather preliminary effectiveness data. This paper reports patient satisfaction with PREVENT and implementation results as well as the preliminary impact on patient motivation, behaviors, and CVH outcomes among a sample of predominantly low-income minoritized adolescent patients aged 12 to 18 years with obesity.

## Methods

### Study Overview

The study took place in a multidisciplinary obesity management clinic at a children’s hospital within an academic medical center. The research team trained clinicians (physicians, nurse practitioners, and dietitians) to use PREVENT and provided on-site support during the trial. PREVENT was used by clinicians in collaboration with patients during their baseline clinical visit. The tool delivers electronic follow-up monthly for 3 months.

### Eligibility and Recruitment

The research team collaborated with a clinical research coordinator embedded within the clinic to identify eligible patients. Patients were eligible for participation if they were aged 12 to 18 years at the time of their scheduled baseline clinic visit, had a BMI ≥85th percentile for their sex and age, spoke English, and were accompanied by a parent or legal guardian (hereinafter referred to as parent) with sufficient English proficiency, were not planning to move out of the clinic service area during the 3-month period after baseline data collection, and did not have severe physical or cognitive limitations that would make physical activity unsafe (as determined by the treating clinician). Patients were excluded if they needed an interpreter during their clinic visit, were not accompanied to the visit by their parent, had a BMI <85th percentile on the day of their clinic visit, or missed their scheduled appointment and did not reschedule within the study period.

The research team used a multimethod recruitment approach. Research assistants (RAs) mailed recruitment letters to eligible patients and their guardians 3 to 6 weeks before their clinic visit and made up to 3 recruitment call attempts. Patients and parents who expressed interest via telephone recruitment were emailed an electronic consent form. The study principal investigator and an RA conducted in-person recruitment of patients who were not successfully contacted before the clinic visit or who had expressed interest but did not complete the electronic consent process.

### Randomization

Once parent consent and minor assent were obtained and verified, patients were randomized to PREVENT or a wait-list control group. We used an alternating assignment approach wherein the first enrolled participant was randomized using a Excel (Microsoft Corp) random assignment function, and subsequent participant assignment alternated between PREVENT and control to achieve balanced group assignment. Blinding was not possible or appropriate for this study because clinicians were aware of which patients were recruited in the clinic and which were assigned to receive PREVENT so that they could plan their workflow accordingly. All enrolled patients completed their routine clinic visit. PREVENT patients also received use of the PREVENT tool, described in the next subsection, with their clinicians during their visit. Waitlist control patients received usual care at the time of their clinic visit and received their PREVENT-generated action plan upon completion of the 3-month follow-up survey.

### PREVENT Intervention

**Figure 1:**
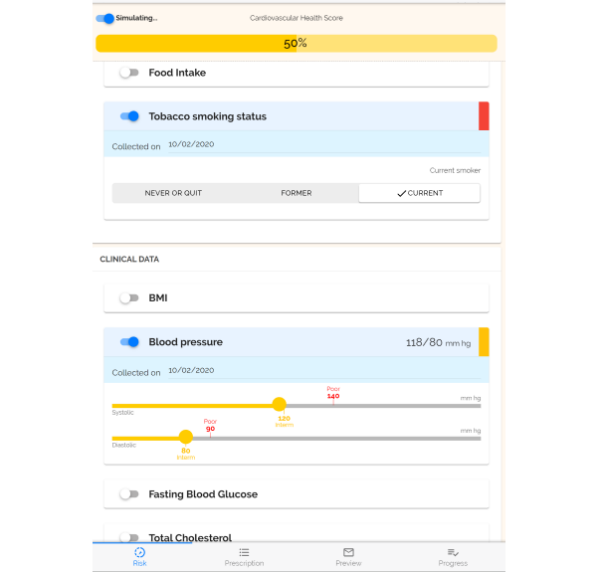
Patient-Centered Real-Time Intervention (PREVENT) tool cardiovascular health profile.

PREVENT is a patient-centered digital health tool designed to improve clinical care as a first-line approach to obesity prevention and management. PREVENT supports health care teams (eg, physicians, nurses, dietitians, and community health workers [CHWs]) in engaging patients in health behavior counseling and action planning. Guided by the AHA *Life’s Simple 7* risk factors and algorithm [5], PREVENT uses patient-reported health behavior (food intake, physical activity, and smoking) and clinical data (height and weight to calculate BMI, blood pressure, fasting blood glucose level, and total cholesterol level) from the EHR to calculate and visually display a CVH score (Figure 1). PREVENT includes color-coded slider bars that allow the clinician to simulate how changes in health behaviors and clinical indicators can impact CVH to educate patients and motivate them to engage in behavior change. PREVENT generates tailored evidence-based goals for physical activity and food intake behavior change based on the patient’s current behaviors and health status. Clinicians can further tailor the goals based on patient needs and preferences; the recommended goals serve as a starting point for shared decision-making discussions between the clinician and the patient to develop behavior change goals and an action plan, including identifying activities and healthy foods that they enjoy and are feasible to include in their lives.

**Figure 2:**
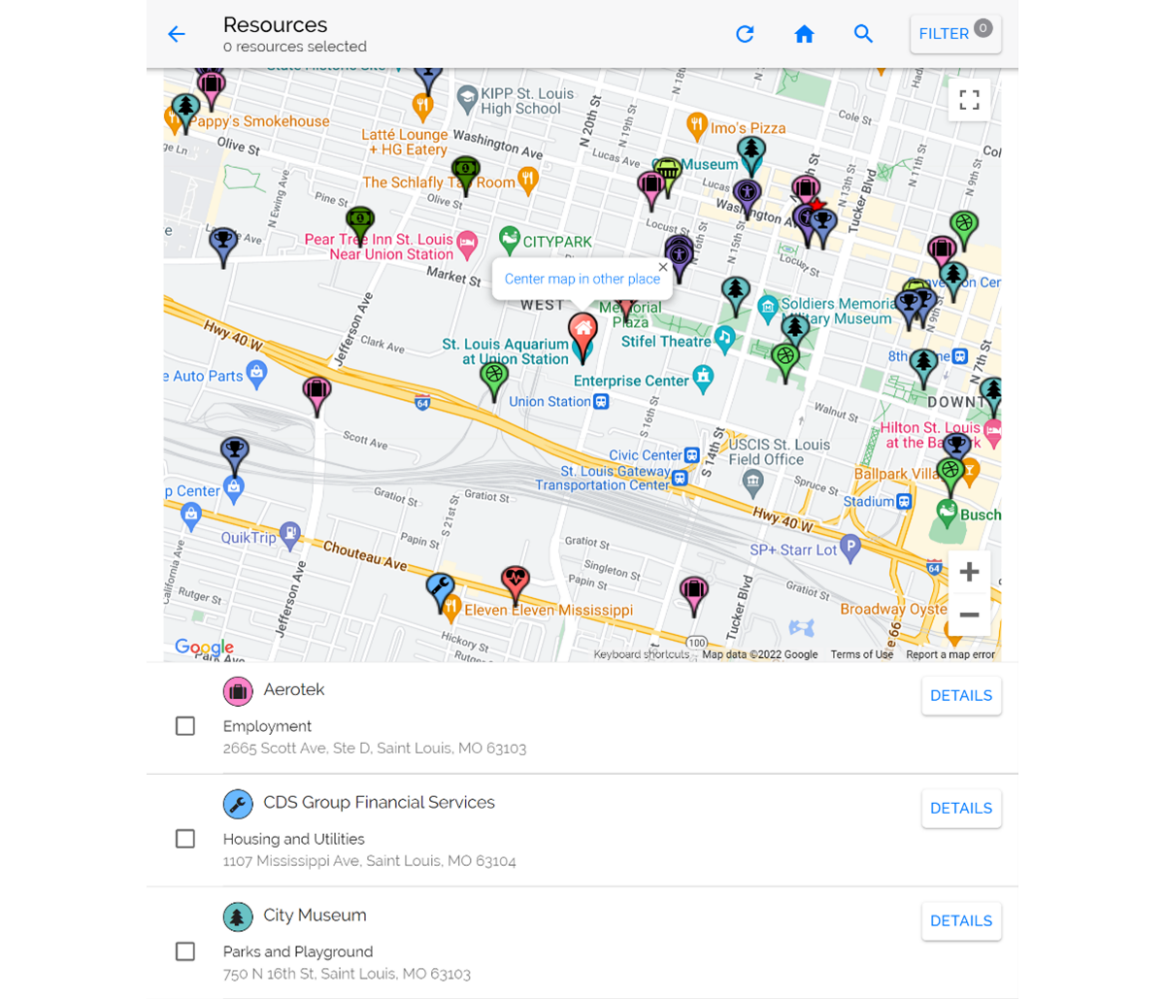
Patient-Centered Real-Time Intervention (PREVENT) tool resource map.

PREVENT also includes a map of community resources (eg, parks, playgrounds, community centers, fitness classes, and farmers’ markets) near a patient’s home (or other preferred address) and a repository of digital resources to allow the patient and clinician to identify health-promoting supports (Figure 2). PREVENT creates a summary action plan that includes the physical activity and nutrition goals, brief educational information on nutrition and physical activity (eg, serving sizes), resource information, and links to the resource map and digital resource repository. This action plan is delivered electronically via email or SMS text message (based on patient communication preferences) from PREVENT and can be printed to include with the after-visit summary. PREVENT also sends monthly automated follow-up emails and SMS text messages, as preferred by the patient, to check on goal attainment. PREVENT delivers new goals if previous goals were met and offers troubleshooting and tailored motivational messages if patients indicate difficulty meeting previous goals. Health care team members can monitor patient progress toward goals on a patient’s dashboard in PREVENT.

A patient’s experiences of autonomy, competence, and relatedness lead to motivation and are affected by health care climates that promote patient autonomy [49,50]. PREVENT seeks to foster this type of environment by (1) providing choices and fostering discussion about what activities or foods a person would like to try to meet their goals (autonomy), (2) demonstrating the value of changing behaviors (autonomy), (3) providing personalized goals that are attainable and resources to support them (competence), and (4) fostering a personalized behavior change discussion with a care team member (relatedness). In this process, a sense of being respected, understood, and cared for is essential to forming the experiences of connection and trust that allow for intrinsic motivation to occur. All health care team members were trained before using the PREVENT tool on the use of neutral language during patient-provider interactions (eg, *may* and *could*, not *should* or *must*) to further support autonomy [51,52].

### Measurement

#### Overview

This study used multiple quantitative and qualitative data collection methods. The study team extracted clinical data from the EHR at baseline and 3-month follow-up. In both groups, patients completed baseline health behavior and household demographic surveys, with parent assistance as needed, administered electronically before, or on the day of, the clinic visit. The study team observed a subset of PREVENT visits, with patient, guardian, and clinician permission, to assess how the PREVENT tool was used during an encounter. At 3-month follow-up, we administered the same health behavior survey, with PREVENT satisfaction questions added for the PREVENT group. We conducted semistructured interviews with a subset of PREVENT patients and their parent who accompanied them to the baseline clinic visit. We recruited all PREVENT participants who completed the 3-month follow-up survey to participate in interviews. We also conducted postintervention surveys and interviews with the health care team members who administered PREVENT (clinician data reported elsewhere [53]). Additional details on the survey measures are included in Multimedia Appendix 1.

#### Demographics

The baseline demographics survey assessed patient date of birth, gender identity, and race and ethnicity. Parent characteristics included biological parent marital status, educational attainment of the patient’s biological mother and father, and health literacy of the parent accompanying the patient to the visit. Household characteristics included household size, income and income stability, food security, neighborhood safety, and transportation reliability. As this study was conducted during the COVID-19 pandemic, we included items on household income and food security changes due to the pandemic.

#### Self-Determination Theory Outcomes

These were assessed in the health behavior surveys delivered at baseline and follow-up. *Motivation* was measured as willingness to change and intrinsic motivation. Patient willingness to change physical activity and food intake was measured using 2 items (one for each behavior) adapted from the Rapid Eating Assessment for Participants survey and rated on a 5-point Likert scale, with higher scores indicating greater willingness [54]. Intrinsic motivation was assessed using a 6-item subscale adapted from existing self-determination theory measures, which includes items assessing intrinsic motivation, defined as acting because the behavior is enjoyable, satisfying, or interesting to the individual [55,56]. *Competence* or one’s perceived ability, measured using the Self-Efficacy for Healthy Eating and Physical Activity measure developed by Steele et al [57] was used to measure the patient’s self-efficacy or competence to engage in specific behaviors related to physical activity and healthy eating [58,59]. *Autonomy* was measured as patient knowledge of CVH risk (perceived value and importance of healthy behaviors), and awareness of resources was assessed using 4 Likert response items, with higher scores indicating greater knowledge.

#### Behavior Change Outcomes

Health behavior surveys delivered at baseline and follow-up assessed physical activity and food intake behaviors. Physical activity questions are from the validated International Physical Activity Questionnaire [60]. Physical activity was reported as minutes per week of moderate and vigorous activity. Food intake questions were based on the Stoplight Diet [61] and Rapid Eating Assessment for Participants questionnaire [54]. Food intake items assessed how frequently (*usually*/*often*, *sometimes*, or *rarely*/*never*) patients met daily intake recommendations for fruits, vegetables, whole grains, sugar-sweetened beverages, and high-sugar snack foods. An overall continuous variable of the sum of food recommendations met was used in the analysis (range: 0-5 food behaviors).

#### CVH Outcomes

Height, weight, fasting blood glucose level, total cholesterol level, blood pressure, and smoking status were extracted from the EHR at baseline and 3-month follow-up, when available. Height and weight were used to calculate BMI and BMI *z* scores based on Centers for Disease Control and Prevention growth charts by sex and age [62]. Using established methods [9,10,63], all CVH metrics from the AHA *Life’s Simple 7* were categorized as poor (0), intermediate (1), or ideal (2) and summed to calculate an overall CVH score (0-14). The score was divided by the total number of available CVH metrics to generate a 0-to-100 percentile score (Multimedia Appendix 2).

#### Satisfaction With PREVENT

Participant satisfaction with PREVENT was assessed via the 3-month follow-up survey and semistructured interviews with a subset of patients and guardians who attended the PREVENT clinic visit. Patients and parents were recruited for interviews by telephone or email depending on their preferred method of contact in the order in which they enrolled. The survey included 5 Likert response items assessing patient perceptions of the PREVENT tool, with higher scores indicating greater satisfaction. The interview questions asked participants to describe their likes and dislikes regarding the tool, how they used information provided by the tool, recommendations for changes or improvements, and their attitudes toward future use of PREVENT with their health care teams. Interviews were conducted until saturation (ie, no new ideas were being heard) was reached.

#### PREVENT Implementation

A subset of baseline clinic visits (n=6) during which PREVENT was used were directly observed by the principal investigator or RA using a standard observation template. The observer timed the duration of PREVENT’s use, including the time spent in each section (CVH risk profile, behavior change prescription, and resource delivery). The observer recorded responses to fixed items assessing clinician use of PREVENT’s features (eg, using slider bars to demonstrate potential impacts of behavior or clinical change on the CVH score and showing the community resource map), key conversation points (eg, explaining physical activity and food intake recommendations), and patient and guardian level of engagement. The observer also wrote open-ended field notes describing PREVENT’s use and any challenges or issues that arose with the tool. Data were downloaded from PREVENT to assess patient engagement with the tool after the visit (eg, the number of times the resource map was opened and responses to automated goal check-in surveys).

### Data Analysis

Distributions of participant characteristics across the 2 groups were analyzed at baseline using 2-tailed *t* tests for continuous variables and chi-square and Fisher exact tests for categorical variables. To examine changes in outcomes from baseline to follow-up, differences were calculated using the difference of the mean (follow-up minus baseline) for continuous variables. Welch unpaired *t* tests were used for within-group significance testing. Differences in mean pre-post changes across the groups were tested using ANOVA for continuous variables (refer to the *Results* section). We performed sensitivity analyses by conducting paired significance testing only with individuals with complete data at baseline and follow-up; the results from this approach did not significantly differ from the results of the unpaired analyses. All analyses were conducted using R software (R Foundation for Statistical Computing).

All qualitative data were professionally transcribed, anonymized, and imported into NVivo 12 (Lumivero) for thematic analysis. Two coders read all transcripts and developed a draft codebook. They pilot-coded 3 transcripts together to refine the codebook, after which they double coded the remaining transcripts and met to generate consensus. Subsequently, the coders generated a table summarizing key themes and illustrative quotes. We triangulated the findings across the survey, observation, and interview data and looked across data sources for convergence, divergence, and explanatory description.

### Ethical Considerations

This pilot randomized feasibility trial was approved by the Washington University in St. Louis Institutional Review Board (IRB 202004230). All participants received an incentive for completing baseline and follow-up measures.

## Results

### Baseline Characteristics

**Figure 3:**
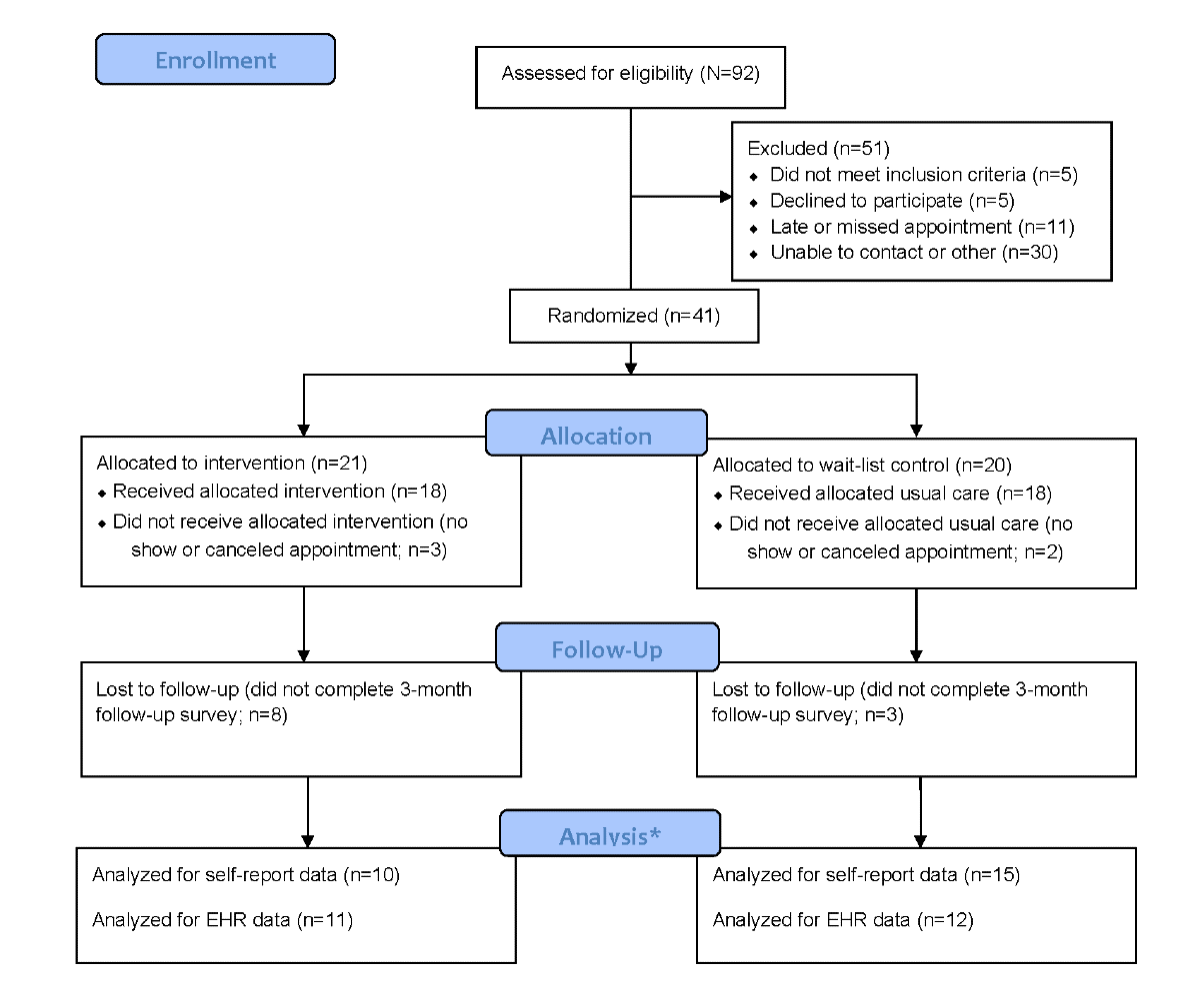
CONSORT (Consolidated Standards of Reporting Trials) flow diagram. *The number of participants included for analysis differs by data source because some patients did not complete the follow-up survey, and some did not have a clinical encounter during the follow-up period from which electronic health record (EHR) data could be extracted.

Of the 92 patients assessed for eligibility, 51 (55%) were excluded for various reasons (n=30, 59% inability to contact the participant or deliver the consent documents; n=11, 22% late arrival or missed appointments; n=5, 10% ineligibility at the time of clinic visit, eg, no accompanying parent or interpretation services required for limited English proficiency; and n=5, 10% declined to participate), and 41 (45%) were randomized (n=21, 51% were randomized to the PREVENT intervention; and n=20, 49% were assigned to wait-list control; Figure 3). Of these 41 patients who were enrolled and randomized before their baseline clinic visit, 5 (12%) missed or canceled their appointment without rescheduling; thus, 36 (88%) patients participated in the trial (n=18, 50% received PREVENT; and n=18, 50% received usual care). All 36 patients completed baseline data collection. In addition, 6 patients and parents completed follow-up qualitative interviews.

Table 1 shows the study sample characteristics, overall (n=36) and by group assignment; the PREVENT and control groups did not significantly differ in demographic characteristics. The average participant age at baseline was 14.72 (SD 1.85) years; 53% (19/36) identified as Black, 33% (12/36) as non-Hispanic White, and 14% (5/36) as ≥1 other races and ethnicities. Of the 36 participants, 7 (19%) lived in households below federal poverty level; 8 (23%) reported unstable family income, and 13 (37%) reported decreased income during the COVID-19 pandemic. Most of the participants had reliable transportation (33/36, 91%), sufficient parent health literacy (30/36, 83%), and resided in safe neighborhoods (32/36, 89%). Of the 36 participants, 14 (40%) came from food-insecure households; 5 (14%) reported decreased food security during the COVID-19 pandemic. Of the 6 interview participants, 4 (67%) identified as Black and 2 (33%) as White; 5 (83%) were girls; and 2 (33%) lived in a household below the poverty level, while 2 (33%) reported decreased household income during the COVID-19 pandemic.

**Table 1:** Baseline characteristics by intervention group.

Adolescent characteristics				Overall (n=36)		Intervention (n=18)		Control (n=18)		*P* value^a^	
Age (years), mean (SD)				14.72 (1.85)		15.00 (1.46)		14.44 (2.18)		.38	
**Gender, n (%)**											.08
	Boy		12 (33)		9 (50)		3 (17)				
	Girl		24 (67)		9 (50)		15 (83)				
**Race, n (%)**											.88
	Black		19 (53)		10 (56)		9 (50)				
	White		12 (33)		6 (33)		6 (33)				
	Other		5 (14)		2 (11)		3 (17)				
**Parent and household, n (%)**											
	**Parental marital status, not married**		19 (53)		9 (50)		10 (59)		.99		
		Missing	1 (3)		N/A^b^		1 (6)				
	**Mother’s education, less than college graduate**		25 (69)		13 (72)		12 (71)		.99		
		Missing	1 (3)		N/A		1 (6)				
	**Father’s education, less than college graduate**		23 (70)		12 (67)		11 (69)		.99		
		Missing	3 (8)		1 (6)		2 (11)				
	Below poverty level		7 (19)		4 (22)		3 (17)		.99		
	**Unstable income**		8 (23)		5 (28)		3 (18)		.69		
		Missing	1 (3)		N/A		1 (6)				
	**Household income decreased during the COVID-19 pandemic**		13 (37)		7 (39)		6 (35)		.99		
		Missing	1 (3)		N/A		1 (6)				
	**Unreliable transportation**		3 (9)		1 (6)		2 (12)		.61		
		Missing	1 (3)		N/A		N/A				
	**Low health literacy**		6 (17)		3 (17)		3 (18)		.99		
		Missing	1 (3)		N/A		1 (6)				
	**Unsafe neighborhood**		4 (11)		2 (11)		2 (12)		.99		
		Missing	1 (3)		N/A		1 (6)				
	**Food insecure**		14 (40)		9 (50)		5 (29)		.31		
		Missing	1 (3)		N/A		1(6)				
	**Food security decreased during the COVID-19 pandemic**		5 (14)		1 (6)		4 (24)		.18		
		Missing	1 (3)		N/A		1 (6)				

^a^*t* test, Pearson chi-square test, or Fisher exact test.

^b^N/A: not applicable.

At baseline, most of the participants (28/36, 78%) had intermediate CVH (overall mean CVH percentile score 54.4 out of 100). Approximately half (20/36, 56%) did not meet physical activity recommendations, and on average participants met 1.64 of 5 (SD 0.96) food intake recommendations. On the 5-point Likert scale, participants indicated low to moderate willingness to change their food intake (mean 2.31) and physical activity (mean 2.22) behaviors and confidence in making these changes (mean 3.94). Participants’ understanding of their CVH was moderate (mean 3.56).

### Changes in Self-Determination Theory Outcomes

At the 3-month follow-up, we obtained survey responses from 25 (69%) of the 36 patients. These participants did not differ from the overall sample. Across both groups, patient willingness to change physical activity and food intake behaviors increased from baseline to follow-up; yet, it did not reach statistical significance within or across the groups (Table 2). Patients in the PREVENT group had a 0.31 mean increase in their intrinsic motivation, whereas those in the control group had a 0.33 mean decrease (overall difference across the groups 0.64). Across both groups, patient physical activity self-efficacy increased by 0.2 from baseline to follow-up; yet, it did not reach statistical significance within or across the groups. For food self-efficacy, the PREVENT group remained the same from baseline to follow-up, whereas the control group showed an increase of 0.3. These changes in food self-efficacy were not significant within or across the groups. Although not significant, patients in the PREVENT group increased their understanding of steps to improve heart health (mean change 0.22), whereas this understanding decreased in the control group (mean change −0.28). The awareness of resources to support behavior change increased in the PREVENT group (mean change 0.54) and decreased in the control group (mean change −0.11); the changes were not statistically significant within or across the groups.

**Table 2:** Changes in motivation, competence, and autonomy from baseline to follow-up within and across the trial groups (all items scored on a 5-point scale).

				Patient-Centered Real-Time Intervention (n=18)						Control (n=18)							Across group difference^a^
				Baseline, mean (SD)		Follow-up, mean (SD)		Within group difference^b^ (95% CI)		Baseline, mean (SD)		Follow-up, mean (SD)		Within group difference^b^ (95% CI)			
**Motivation**																	
	**Willingness to change physical activity**		2.11 (1.13)		2.56 (0.88)		0.44 (–0.38 to 1.27)		2.33 (1.24)		3.07 (0.96)		0.73 (–0.05 to 1.51)		–0.29		
		Missing, n	0		9		N/A^c^		0		3		N/A		N/A		
	**Willingness to change food intake**		2.22 (1.31)		3.00 (1.05)		0.78 (–0.16 to 1.72)		2.39 (1.46)		3.07 (0.68)		0.70 (–0.19 to 1.55)		0.10		
		Missing, n	0		8		N/A		0		3		N/A		N/A		
	**Intrinsic motivation**		3.49 (0.97)		3.80 (0.82)		0.31 (–0.41 to 1.03)		3.80 (0.47)		3.48 (0.77)		–0.33 (–0.80 to 0.15)		0.64		
		Missing, n	0		8		N/A		1		3		N/A		N/A		
**Competence**																	
	**PA self-efficacy**		3.47 (0.66)		3.71 (0.72)		0.24 (–0.37 to 0.85)		3.54 (0.88)		3.73 (0.83)		0.19 (–0.43 to 0.81)		0.05		
		Missing, n	0		9		N/A		1		3		N/A		N/A		
	**Food self-efficacy**		3.56 (0.94)		3.57 (0.69)		0.01 (–0.65 to 0.68)		3.51 (0.83)		3.83 (0.80)		0.31 (–0.28 to 0.90)		–0.30		
		Missing, n	0		9		N/A		1		3		N/A		N/A		
**Autonomy**																	
	**Understand risk for poor heart health**		3.67 (0.97		3.70 (1.06)		0.03 (–0.82 to 0.89)		3.78 (0.81)		3.87 (0.99)		0.09 (–0.57 to 0.74)		–0.06		
		Missing, n	0		8		N/A		0		3		N/A		N/A		
	**Understand steps to improve heart health**		3.78 (0.94)		4.00 (1.15)		0.22 (–0.69 to 1.13)		3.94 (1.06)		3.67 (0.98)		–0.28 (–1.00 to 0.44)		0.50		
		Missing, n	0		8		N/A		0		3		N/A		N/A		
	**Awareness of resources**		3.06 (1.21)		3.60 (1.17)		0.54 (–0.43 to 1.52)		3.11 (1.13)		3.00 (1.20)		–0.11 (–0.95 to 0.72)		0.65		
		Missing, n	0		8		N/A		0		3		N/A		N/A		

^a^Difference in mean within-group difference (intervention mean difference minus control mean difference); ANOVA *t* test.

^b^Welch unpaired *t* test.

^c^N/A: not applicable.

### Changes in CVH Behaviors and Outcomes

At baseline, all patients had BMI and blood pressure data available in the EHR; 75% (27/36) also had cholesterol data available, while 72% (26/36) had blood glucose data available. At the 3-month follow-up, of the 36 patients, 24 (67%) had BMI and blood pressure data available, 6 (17%) had cholesterol data available, and 8 (22%) had blood glucose data available. Total cholesterol and blood glucose data are presented in Table 3 to examine trends, but due to a large amount of missing data, statistical significance tests are not reported. Overall changes in CVH percentile significantly differed across the groups (*P*=.01), with a significant improvement in the PREVENT group (*P*=.02) but not in the control group (*P*=.10). The BMI *z* score decreased in the PREVENT group (mean −0.06) as well as in the control group (mean −0.04); these changes were not statistically significant. Changes in systolic blood pressure significantly differed across the groups (*P*=.001), with significant improvement in the PREVENT group (*P*=.009) but not in the control group (*P*=.31). Likewise, diastolic blood pressure significantly improved in the PREVENT group (*P*=.009) but not in the control group (*P*=.20); yet, these differences were not significantly different across the groups (*P*=.43). Blood glucose level decreased in the PREVENT group (mean −8.38) as well as in the control group (mean −0.09); these changes were not statistically significant. Moderate and vigorous physical activity minutes decreased in the PREVENT and control groups; these changes were not significant. The number of food recommendations slightly decreased in the PREVENT and control groups; these changes were not significant.

**Table 3:** Changes in cardiovascular health (CVH) behaviors and outcomes from baseline to follow-up within and across intervention groups.

				Patient-Centered Real-Time Intervention (n=18)							Control (n=18)							Across group difference^a^
				Baseline, mean (SD)		Follow-up, mean (SD)		Within group difference^b^ (95% CI)		Baseline, mean (SD)			Follow-up, mean (SD)		Within group difference^b^ (95% CI)			
**CVH outcomes**																		
	**CVH percentile^c^**			52.94 (9.67)		63.62 (14.09)		10.68 (2.06 to 19.30)^d^		55.89 (10.93)			50.28 (11.6)		–5.61 (–13.25 to 2.03)		16.29^d^	
		Missing, n	0		2		N/A^e^		0			0		N/A		N/A		
	**BMI *z* score**			2.59 (0.32)		2.54 (0.34)		–0.06 (–0.31 to 0.20)		2.42 (0.26)			2.38 (0.30)		–0.04 (–0.3 to 0.2)		–0.02	
		Missing, n	0		6		N/A		0			6		N/A		N/A		
	**Systolic blood pressure (mm Hg)**			126.06 (9.16)		115.33 (10.53)		–10.72 (–18.46 to –2.98)^f^		123.11 (8.84)			128.17 (14.88)		5.06 (–5.06 to 15.17)		–15.78^f^	
		Missing, n	0		6		N/A		0			6		N/A		N/A		
	**Diastolic blood pressure (mm Hg)**			79.28 (4.39)		71.75 (7.92)		–7.53 (–12.86 to –2.19**)**^f^		81.11 (6.44)			76.42 (10.85)		–4.69 (–12.07 to 2.68)		–2.84	
		Missing, n	0		6		N/A		0			6		N/A		N/A		
	**Total cholesterol (mg/dL)**			159.80 (30.56)		165.50 (35.73)		5.70 (–46.92 to 58.32)		167.59 (29.48)			179.00 (49.50)		11.41 (–366.45 to 389.27)		–5.71^g^	
		Missing, n	8		14		N/A		1			16		N/A		N/A		
	**Blood glucose (mg/dL)**			99.38 (31.14)		91.00 (10.54)		–8.38 (–36.83 to 20.08)		94.89 (16.54)			94.80 (17.58)		–0.09 (–21.46 to 21.29)		–8.29^g^	
		Missing, n	10		15		N/A		0			13		N/A		N/A		
**CVH behaviors**																		
	**Moderate physical activity (minutes/week)**			215.28 (250.90)		153.75 (149.95)		–61.53 (–226.44 to 103.39)		190.00 (204.05)			122.73 (183.20)		–67.27 (–218.75 to 84.20)		5.74	
		Missing, n	0		10		N/A		0			7		N/A		N/A		
	**Vigorous physical activity (minutes/week)**			105.00 (142.67)		94.00 (126.27)		–11.00 (–119.64 to 97.64)		64.17 (136.60)			28.08 (65.75)		–36.09 (–112.17 to 39.99)		25.09	
		Missing, n	0		8		N/A		0			5		N/A		N/A		
	**Number of food recommendations met**			1.72 (0.89)		1.67 (1.50)		–0.06 (–1.25 to 1.14)		1.56 (1.04)			1.33 (0.98)		–0.22 (–0.94 to 0.50)		0.16	
		Missing, n	0		9		N/A		0			3		N/A		N/A		

^a^Difference in mean within-group difference (intervention mean difference minus control mean difference); ANOVA *t* test.

^b^Welch unpaired *t* test.

^c^CVH percentile (range 0-100) is calculated using a published algorithm using all available data from the 7 CVH risk factors. Each risk factor is scored using 3 criteria (0=poor, 1=intermediate, and 2=ideal), summed and divided by the number of variables included to generate a percentile.

^d^*P*<.05.

^e^N/A: not applicable.

^f^*P*<.001.

^g^Statistical test was not conducted due to high proportion with missing data.

### Implementation of PREVENT

Clinicians spent 4 to 11 minutes and, on average, 6.5 minutes using PREVENT with patients. Approximately 2.3 minutes were spent discussing patient CVH, 2.5 minutes delivering health behavior goals, and 1.8 minutes discussing resources. Patients most frequently requested information on grocery stores, recreation centers, parks, playgrounds, and nutrition-related digital resources. On average, patients received information about 6.5 resources. Most of the patients and parents were moderately to very engaged when using PREVENT with their clinician per observer ratings. Although the PREVENT tool includes automated monthly check-ins on patient goals, only a few PREVENT patients (4/18, 22%) engaged with these surveys. Observation data indicate that clinicians did not remind patients to complete the goal check-ins. The observations also uncovered a technical issue: simultaneous users logged into the same patient profile were unable to view the other user’s updates (eg, a physician logged into a patient’s PREVENT profile at the same time as a dietitian did not see adjustments made to health behavior goals until the other user logged out). The web developers working with the study team were able to fix this issue during the trial.

### Patient Satisfaction With PREVENT

Of the 18 PREVENT patients, 10 (56%) completed the follow-up satisfaction items, and we conducted 6 interviews (n=3, 50% with parents; and n=3, 50% with patients) to ascertain what contributed to satisfaction with PREVENT. Overall, patients were moderately satisfied with PREVENT (mean 3.8, SD 0.1). Patients indicated that the tool “helped [them] quite a bit,” and parents noted that they “got more information” than they typically would at a routine visit. Patients found it helpful to see their risk for poor heart health (mean 4.0, SD 0.7). One patient noted as follows:

It was helpful to tell me...not just to think about my eating but to watch out for other things.

Interview participants noted that the data visualizations were helpful. One patient stated as follows:

It’s helpful to have the visuals...I can see what’s going on with me and understand it.

Patients found the recommendations for behavior change moderately easy to understand (mean 3.9, SD 0.7). Participants indicated that the CVH, physical activity, and food intake information was easy to understand, and they appreciated receiving information “in plain English” rather than medical jargon. One parent stated that using PREVENT with their child’s clinician was “quick and easy and didn’t require a lot of extra effort.” Patients and parents expressed that the food recommendations helped them to identify target food intake habits to change (eg, cutting back on sugar sweetened beverages) and healthier options to incorporate. Patients also liked having specific suggestions for feasible activities that they could incorporate into their routine to be more active, although physical activity recommendations were used less than the dietary recommendations.

Patients reported that it was moderately helpful to receive resources (mean 3.8, SD 0.9). One patient shared as follows:

[The resource information helped with] adjusting my daily routine and all the options...fun stuff I could do...gave me many different ideas.

Patients and parents offered suggestions for improvements to the resource features; for example, a parent suggested that “it would be neat to be able to access it [PREVENT] through an app.”

A parent of a family living in a rural area noted the limitation of the resource map for their community, reflecting as follows:

We live in a very small community where there’s not a whole lot to do...if you make that radius a little wider...and you encompass the town that’s 15 to 20 minutes away from us, you’d get that recreation center...if you put that in our zip code, you’re not going to pick anything up.

Another suggestion included adding resources to identify healthy food options at restaurants or places for physical activity while traveling out of the family’s home area (eg, locations for active recreation while on vacation).

Overall, patients were in favor of their clinician using PREVENT in future clinic visits (mean 3.8, SD 0.6). All interview participants indicated that they would want to use the tool again at a future visit. One parent shared as follows:

I was pretty satisfied with the appointment and PREVENT put everything up that we talked about...I wouldn’t want to change a thing.

Other parents expressed hesitancy around conversations focused on weight and BMI and were appreciative that other indicators, such as blood pressure, and behaviors were used.

## Discussion

### Principal Findings

In this pilot randomized feasibility trial, we found that the PREVENT intervention resulted in modest improvements in patients’ motivation to change, their autonomy (ie, perceived control), and overall CVH score. Importantly, the PREVENT intervention was feasible to deliver within a routine clinical care visit and was acceptable to patients and families. This trial demonstrates the ability to recruit and randomize participants from the target population and deliver our intervention to them. Furthermore, this trial demonstrates patient and parent satisfaction and acceptance, which predicts key outcomes such as user engagement, intervention effectiveness, and widespread adoption. Our team gained insight on PREVENT’s features and uses and received suggestions for improvement that may help increase satisfaction and usefulness in future studies [64]. In addition to its acceptance by patients, the success of PREVENT relies on fit within the intended context (eg, clinic workflows, time frames, and resources). Our observational data revealed an issue with simultaneous users that limited PREVENT’s usability in a team care environment; this was successfully resolved in this pilot feasibility trial. This trial is a critical step that has informed necessary changes in a digital health intervention to improve care delivery for patients with overweight and obesity. We will use insights from this trial to design and conduct a subsequent efficacy trial before conducting a fully powered clinic-randomized effectiveness trial.

Health behavior counseling delivered using PREVENT resulted in positive changes in patients’ intrinsic motivation and autonomy. An understanding of the necessary steps needed to improve one’s health builds perceived control (autonomy), which the self-determination theory posits is a critical step to initiating and maintaining behavior change. The chronic care model emphasizes the importance of an informed, activated patient [18-20]. The PREVENT tool demonstrated suitability for engaging patients in their care, which supports patient autonomy and self-determination, promotes confidence and trust in the clinician-patient relationship, and improves satisfaction with care [18-20]. Patients and parents liked having data visualizations to accompany the patient-clinician conversation. PREVENT is one of the few available tools, designed to support the health care team, that include interactional features, data visualization, and evidence-based approaches to deliver behavior change goals that are tailored to the patient. A major strength was the use of digital features to promote self-determination theory principles that resulted in increased motivation among the intervention patients. Increasing intrinsic motivation is linked to the completion of behavior change interventions and is important for the promotion and sustainment of physical activity and healthy food intake behaviors among children and adolescents [65,66].

The delivery of tailored goals and changes in patients’ motivation and autonomy did not translate to detectable increases in self-reported physical activity and food intake behaviors, although this feasibility trial was not powered to determine effectiveness as a primary outcome. In the subset of observed intervention visits, clinicians did not mention the automated goal check-ins to patients, and only a few patients responded to PREVENT’s automated goal check-ins sent via email or SMS text message. Patients may have missed out on reminders and tailored encouragement messages for achieving their health behavior goals, reducing the intended dose of the intervention. Furthermore, this lack of continued engagement may have contributed to the overall low response rate at 3 months. Learnings from this pilot trial offer insights for improving training and ongoing reminder strategies for health care teams to emphasize the goal check-ins and deliver automated reminders to encourage patients to complete goal check-ins that would further patient contact. To further improve health behavior counseling, PREVENT may need to be implemented by a care team member with more time to work with the patient to set goals, deliver resources, and follow up with patients. In addition, PREVENT may be expanded to incorporate self-monitoring tools (eg, activity trackers such as Fitbit devices and food logs) that increase the accuracy and frequency of contacts and feedback. In the current setting, clinicians spent an average of 6.5 minutes delivering PREVENT at each visit. Additional care team members, such as CHWs, may be well suited to deliver PREVENT’s goal and resource information. CHWs’ unique rapport and cultural congruence with patients make them ideal members of the health care team to help patients adopt healthy behaviors and address unmet social needs [67]. CHW-led counseling can result in behavior change, but, to our knowledge, it has not been previously supported by a digital health tool such as PREVENT [68-70].

Promoting health behavior change within clinical workflow is impacted by multilevel factors outside the clinic [11]. A strength of this trial was the inclusion of a diverse patient population with high levels of social needs (eg, food insecurity, unstable income, and low parent health literacy). In addition, this study demonstrates the feasibility of providing information on resources to support physical activity and healthy food intake within a clinic visit using a digital health tool (PREVENT). Patients who received the PREVENT intervention modestly increased their awareness of health-promoting resources and indicated that it was helpful to receive such resource information. Further work is needed to understand the use of resources delivered via PREVENT and the relation to changes in behaviors and CVH outcomes. Several other primary care–based interventions have linked patients to community resources and show promising impacts on weight loss in adults and children [37-39,71]. Nevertheless, these studies did not use a digital health approach that may allow for widespread adoption and dissemination.

This study demonstrated the feasibility of using a digital approach to facilitate health behavior counseling, inclusive of tailored goals and community resources, and this aligns with behavior change theory. This study also provides meaningful insights into recruiting participants and collecting data to examine effectiveness within a routine care setting.

As in any pilot feasibility study, this study has several limitations. First, the sample size is small and was not powered to detect clinical changes, although we analyzed patient behavior and clinical data to detect a signal for change that will be used to inform a sufficiently powered trial to test the effectiveness of PREVENT. Second, the study was conducted in a specialty obesity clinic at a large academic medical center and may not be representative of other pediatric practices treating patients with overweight and obesity. This clinic specializes in obesity management and has resources (eg, staff and extended visit lengths) that support the delivery of behavior change counseling beyond those often found in primary care clinics. This may have diminished differences across the groups because even control patients in routine care received counseling on physical activity and nutrition. Our team is conducting additional pilot trials of PREVENT in other clinical settings to determine whether similar patterns in patient motivation, behavioral, and clinical outcomes are observed. Other studies will also expand on the qualitative and direct observation findings that examined implementation in a small subset of patients and parents. Our team is currently collaborating with a rural federally qualified health center network serving a geographic area that has higher rates of obesity and poorer health behaviors than US averages to plan for additional feasibility and preliminary effectiveness testing in more resource-limited environments. These ongoing trials offer opportunities to improve training materials and implementation plans based on pilot findings. PREVENT is focused on the critical first step of improving health behavior counseling to motivate patients. Behavioral science has demonstrated the need for frequent contact and self-monitoring for maintaining healthy behaviors. Of particular focus in future trials is using other care team members (eg, CHWs) to improve patient follow-up as well as increase self-monitoring. In addition, patients who require more intensive intervention and support (eg, those who are severely obese or those not willing to change) may be referred to other interventions or programs that are listed in PREVENT’s resource map and library.

The short 3-month follow-up period of this pilot trial may not have allowed for adequate time to observe changes in behaviors or clinical outcomes. CVH data in the EHR were often not available at the 3-month follow-up because patients typically visit the clinic every 6 months or annually, and not all patients have EHR data shared from other care sources (eg, primary care physician or urgent care). This feasibility study generates an understanding of the ability to capture the AHA *Life’s Simple 7* (now *Life’s Essential 8*) metrics using routine care clinical data. Based on frequency at baseline and follow-up in this population, we conclude that BMI, blood pressure, and smoking status are the most common metrics assessed**,** whereas cholesterol and blood glucose tests are obtained less frequently, which aligns with clinical care guidelines for patients with prediabetes. This may warrant multiple and longer follow-up times in future studies, especially because cognitive precursors to behavior change and behavior changes themselves may precede changes in clinical indicators observed only after longer periods. Furthermore, this study relied on self-report behavioral data, which are prone to bias [72,73]. Although we attempted to collect objective data, the COVID-19 pandemic presented challenges that required changes to our data collection procedures and may have influenced patient ability to engage in health behavior change or to access health-promoting resources. Furthermore, the COVID-19 pandemic may have created competing priorities or demands that contributed to our low retention rate of 69% (25/36 completed surveys at the 3-month follow-up). The lack of continued engagement throughout the 3-month period noted earlier may have also contributed. Follow-up data collection relied entirely on electronic surveys; yet, extending the follow-up period to align with routine care may have allowed for in-person data collection at a subsequent clinic visit. Future trials can capitalize on creative solutions for patient retention and data collection emerging from other research to improve retention rates and the use of objective measures such as accelerometers and digital scales.

### Conclusions

This study offers valuable insights for testing digital tools to support behavior change counseling in clinical settings. Such tools hold promise for supporting shared decision-making among patients and clinicians and improving the communication of CVH information to patients. Digital health tools that align with care goals and quality metrics can be incorporated into clinical workflows with minimal time needed and can enhance recommended counseling on weight management and obesity prevention. Efforts are underway to improve the PREVENT tool based on lessons learned from this trial and to develop implementation plans that align with clinic workflows to optimize implementation, further understand impacts on patient behaviors and CVH outcomes, and expand to more generalizable clinic settings.

## Appendix

Multimedia Appendix 1 Survey measures.

Multimedia Appendix 2 Cardiovascular health score.

## Abbreviations

AHA: American Heart Association
CHW: community health worker
CVH: cardiovascular health
EHR: electronic health record
PREVENT: Patient-Centered Real-Time Intervention
RA: research assistant
