# Supplementary material for: The Impact of Behavior Change Counseling Delivered via a Digital Health Tool Versus Routine Care Among Adolescents With Obesity: Pilot Randomized Feasibility Study
Source: JMIR Form Res. 2024 May 17;8:e55731. doi: 10.2196/55731 (PMC11143394; doi:10.2196/55731)
Supplement: Multimedia Appendix 1 [file formative_v8i1e55731_app1.docx]

**Supplemental File 1: Measures Overview**

| **Survey** | **Construct** | **Measure Source** | **# of items used** | **Response Options/Scale** | **Example Item** |
| --- | --- | --- | --- | --- | --- |
| Patient and Family Demographics | Gender | Team-generated | 1 | Male, female, non-binary/third gender, transgender, prefer to self-describe | *What is your gender? (Separate items for patient and guardian)* |
|  | Race | Team-generated | 1 | American Indian or Alaska Native, Asian, Black or African American, Hawaiian or Pacific Islander, White, Middle Eastern/North African, Multi-Race, Other | *What race do you consider yourself to be? (Separate items for patient and guardian)* |
|  | Ethnicity | [BRFSS](https://www.cdc.gov/brfss/questionnaires/pdf-ques/2020-BRFSS-Questionnaire-508.pdf) | 1 | Hispanic (Latino/Latina), Non-Hispanic | *Are you of Hispanic, Latino, or Spanish origin?* |
|  | Child grade-level | Team-generated | 1 | 6^th^, 7^th^, 8^th^, 9^th^, 10^th^, 11^th^, 12^th^, other | *What grade is your child in? (if between grades, select the grade that they just completed)?* |
|  | Parent education | Team-generated | 1 | No high school degree, some high school, high school diploma/ GED, Associate’s degree or 1-3 years of college, Bachelor’s degree, Graduate/professional degree | *What is the child’s biological mother’s/father’s highest level of education completed? (separate items for each biological parent)* |
|  | Parent marital status | Team-generated | 1 | Married, divorced or separated, never married, widowed, parent unknown | *What is the marital status of the child’s biological parents?* |
|  | Household size | Team-generated | 1 | Numerical entry | *How many people are in your household? (include yourself in this number - for example, if household includes you, a spouse, and two children, the answer is 4)* |
|  | Household income | [BRFSS](https://www.cdc.gov/brfss/questionnaires/pdf-ques/2020-BRFSS-Questionnaire-508.pdf)* | 1 | Less than $10,000, $10,000 - $29,999, $30,000 - $ 49,999, $50,000 - $69,999, $70,000 - $89,999, $90,000 - $109,999, $110,000 - $139,999, $140,000 and above | *What is the combined annual household income for your household (before taxes)? This includes all supplemental income including governmental subsidies.* |
|  | Household income stability | [Daniels & Grinstein-Weiss, 2019](https://papers.ssrn.com/sol3/papers.cfm?abstract_id=3293988); Team-generated | 4 | Fixed response, varies by item | *Which of the following best describes your household's income over the last 6 months?* |
|  | Food security | [Hager et al., 2010](https://pubmed.ncbi.nlm.nih.gov/20595453/); [Your Current Life Situation (YCLS)](https://sdh-tools-review.kpwashingtonresearch.org/screening-tools/your-current-life-situation); Team-generated | 4 | Fixed response, varies by item (yes/no for example) | *Are you easily able to get enough food to eat?* |
|  | Health literacy | Your Current Life Situation (YCLS) | 1 | Yes/no | *Are you easily able to get enough healthy food to eat?* |
|  | Food security | Accountable Health Communities Health-Related Social Needs Screening Tool  (AHC-HSRN) | 10 | (Varies by item) select one | *Within the past 12 months, how often were you worried that your food would run out before you got money to buy more?* |
|  | Transportation needs |  |  |  |  |
|  | Perceived neighborhood safety | Youth Risk Behavior Surveillance System (YRBSS) | 1 | Likert (1 = Never, 5 = Always) | *How often do you feel safe and secure in your neighborhood?* |
| Patient Health Behavior | Physical Activity | International Physical Activity Questionnaire (IPAQ) | 4 | Numerical entry 1-7 days or select “none”, numerical entry hours and minutes | *During the last 7 days, on how many days did you do vigorous physical activities like heavy lifting, digging, aerobics, or fast bicycling? How many minutes per day* |
|  | Food Intake | Stoplight Diet and Rapid Eating Assessment for Participants - Shortened Version (REAPS) | 5 | Yes/no | *Do you eat 3 or more servings of whole grains daily?* |
|  | Willingness to change behavior | REAPS | 2 | Likert (1 = Very interested, 5 = Not at all interested) | *How interested are you in making changes in your eating habits in order to be healthier?* |
|  | Patient knowledge | Team-generated | 6 | Likert (1 = Strongly agree, 5 = Strongly disagree) | *I am confident I understand my risk for poor heart health.* |
|  | Motivation to change | Treatment Self-Regulation Scale | 11 | Likert (1 = Not true at all, 5 = Very true) | *I personally believe that these are important in remaining healthy.* |
|  | Self-Efficacy | Self-Efficacy for Healthy Eating and Physical Activity (SE-HEPA) | 16 | Likert (1 = Disagree a lot, 5 = Agree a lot) | *I can be physically active during my free time on most days* |
|  | Patient satisfaction with PREVENT (Intervention Post-Only) | Team generated | 5 | Likert (1 = Strongly agree, 5 = Strongly disagree) | *I liked the tool my provider used during my last clinic visit.* |
| Goal Attainment Survey | Physical activity | ? | 1 |  |  |
|  | Food intake | ? | 5 |  |  |

*Denotes item(s) adapted from original source
