# Supplementary material for: The Impact of Behavior Change Counseling Delivered via a Digital Health Tool Versus Routine Care Among Adolescents With Obesity: Pilot Randomized Feasibility Study
Source: JMIR Form Res. 2024 May 17;8:e55731. doi: 10.2196/55731 (PMC11143394; doi:10.2196/55731)
Supplement: Multimedia Appendix 2 [file formative_v8i1e55731_app2.pdf]

PREVENT STUDY'S

CARDIOVASCULAR HEALTH SCORE

FOR ADOLESCENTS 12 TO 19 YEARS OF AGE

Based on patients' EHR and self-reported survey data, the following 7 factors will be assessed, categorized, and scored as follows:

The American Heart Association's

LIFE'S SIMPLE 7	PHYSICAL ACTIVITY (MINS/DAY)	HEALTHY FOOD INTAKE BEHAVIORS	BODY MASS INDEX (PERCENTILE)	FASTING BLOOD GLUCOSE	TOTAL CHOLESTEROL	BLOOD PRESSURE (PERCENTILE)	SMOKING STATUS (TOBACCO)	POINTS
POOR HEALTH	0	0-1	> 95th	≥ 126 mg/dL	≥ 200 mg/dL	> 95th	Current	0
INTERMEDIATE HEALTH	1-59	2-3	85th - 95th	100 - 125 mg/dL	170-199 mg/dL	90th - 95th	Former < 12 mos	1
IDEAL HEALTH	≥ 60	4-5	< 85th	< 100 mg/dL	< 170 mg/dL	< 90th	Never/ quit > 12 mos	2

CALCULATION OF TOTAL CVH SCORE

$$\frac{\text{Sum of all factor scores}}{\text{Total possible number of points (max 14)}} = \text{Total CVH Score}$$

The overall CVH score is categorized as poor, intermediate, or ideal health based on the following ranges:

POOR HEALTH < 50%: 1, 2, 3, 4, 5, 6, 7

INTERMEDIATE HEALTH 50% TO 72%: 8, 9

IDEAL HEALTH > 72%: 10, 11, 12, 13, 14

PREVENT STUDY'S

CARDIOVASCULAR HEALTH SCORE

FOR ADOLESCENTS 12 TO 19 YEARS OF AGE

EXAMPLE PATIENT

The American Heart Association's

LIFE'S SIMPLE 7	PHYSICAL ACTIVITY (MINS/DAY)	HEALTHY FOOD INTAKE BEHAVIORS	BODY MASS INDEX (PERCENTILE)*	FASTING BLOOD GLUCOSE	TOTAL CHOLESTEROL	BLOOD PRESSURE (PERCENTILE)**	SMOKING STATUS (TOBACCO)	
EXAMPLE PATIENT DATA	30	1	86th	—	—	94th	Never	
POINTS	1 +	0 +	1 +	0 +	0 +	1 +	2 =	5

EXAMPLE PARTICIPANT CVH SCORE

sum of all factor points

10 instead of 14 due to the unknown data

$$\frac{5}{10} = 50^{th}$$

percentile

POOR HEALTH
< 50%

INTERMEDIATE HEALTH
50% TO 72%

IDEAL HEALTH
> 72%

1 2 3 4 5 6 7 8 9 10 11 12 13 14

*percentiles calculated using the Centers for Disease Control and Prevention growth charts for the United States

** percentiles calculated according to age and height
